# Distribution Dynamics of Wide‐Ranged and Narrow‐Ranged Species From the Pliocene to the Future: Insights From Asian Endemic *Holcoglossum* (Orchidaceae)

**DOI:** 10.1002/ece3.71301

**Published:** 2025-04-14

**Authors:** Pei‐Yang Zhang, Peng Zhou, Yi‐Zhen Liu, Zhi‐Wen Xu, Lei‐Qiang Gong, Xiao‐Guo Xiang

**Affiliations:** ^1^ Key Laboratory of Poyang Lake Environment and Resource Utilization Ministry of Education, School of Life Sciences Nanchang University Nanchang Jiangxi China; ^2^ Jiangxi Poyang Lake Wetland Conservation and Restoration National Permanent Scientific Research Base, National Ecosystem Research Station of Jiangxi Poyang Lake Wetland Nanchang University Nanchang Jiangxi China; ^3^ Administration Bureau of Poyang Lake National Nature Reserve in Jiangxi Province Nanchang Jiangxi China

**Keywords:** climate change, *Holcoglossum*, MaxEnt, narrow‐ranged species, potential distribution, wide‐ranged species

## Abstract

Climate change is an important driver of the potential distribution changes of plants. However, the potential distribution changes of wide‐ranged and narrow‐ranged species in response to climate change were still controversial. An epiphytic orchid genus *Holcoglossum* is a key group to address this issue, with about 30% of species widely distributed in the Asian mainland, while the others are only narrowly distributed in special mountains. Combining with species' occurrences, the environmental variables, and Human Footprint data, we analyzed the key predictor variables and predicted the potential distributions and centroid shifts of four wide‐ranged and four narrow‐ranged *Holcoglossum* species from the Pliocene to the future using the maximum entropy (MaxEnt) model. Our results showed that the potential distributions of seven *Holcoglossum* species (except *H. subulifolium*) were mainly impacted by the precipitation of the warmest quarter in the future. From the Pliocene to the present, the potential distributions of the wide‐ranged species (except *H. subulifolium*) and the narrow‐ranged species were contracted. From the present to the future (SSP2‐4.5, 2090), the potential distributions of two wide‐ranged species ( *H. flavescens* , *H. himalaicum*) would contract, whereas the other two would expand; the potential distributions of two narrow‐ranged species (*H. kimballianum*, *H. wangii*) would contract, and the other two would expand. The centroids of three wide‐ranged species would migrate southwards (*H. amesianum*, *H. himalaicum*, and *H. subulifolium*), whereas *H. flavescens* would have nearly no migration; the centroids of three narrow‐ranged species would migrate southwards ( *H. pumilum* , *H. quasipinifolium*, and *H. wangii*), whereas *H. kimballianum* would migrate westwards. We found that the vulnerability to climate change of species might be unlinked to their current distribution range and the phylogenetic relationships. This study provides new insights for the potential distribution changes and conservation of narrow‐ranged and wide‐ranged orchid species.

## Graphical abstract

The potential distributions were mainly impacted by the precipitation of the warmest quarter. From the Pliocene to the future, the potential distributions of both wide‐ranged and narrow‐ranged *Holcoglossum* species were predicted to be contracted or expanded. The centroids of most wide‐ranged *Holcoglossum* species would shift westwards in the future, whereas most narrow‐ranged species would migrate to the lower latitudes.

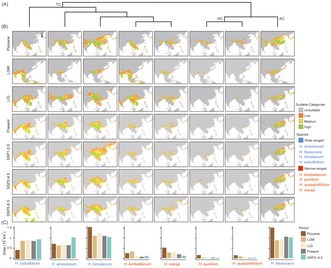

## 1 Introduction

Species migrations in response to climate change may significantly influence local biodiversity loss, structures, and functions of ecosystems (Mantyka‐Pringle et al. 2015). With global warming in the future, the potential distributions of some plants will expand, such as *Angelica dahurica* (Zhang et al. 2024), *Lycium barbarum* (Li et al. 2024), and *Chamorchis alpina* (Kolanowska et al. 2024), whereas others will contract, such as *Dendrohydrax lindenii* (Kolanowska 2023), *Ophrys insectifera* (Charitonidou et al. 2022), and most *Oreocnide* species (Wu et al. 2022). In addition, species with different current distribution ranges seem to have different response patterns to climate change in the future. For example, the potential distribution areas of wide‐ranged *Causonis* species (e.g., *C. japonica* ) would expand in the future, whereas the area of narrow‐ranged species (e.g., *C. ciliifera*) would contract (Yu et al. 2023). A hypothesis was put forward that narrow‐ranged species were considered to be more vulnerable to climate change and at higher risk of extinction than wide‐ranged species (Purvis et al. 2000; Yu et al. 2017; Newbold et al. 2018; Xu et al. 2019; Wang et al. 2022). This hypothesis was supported in some research, for example, Wang et al. (2022) found that the vulnerability of *Magnolia* species under future climate change was negatively related to their current range size. However, the potential distributions of some narrow‐ranged species would also expand, such as Rhododendrons (Yu et al. 2019) and some *Calanthe* species (Qiu et al. 2023). Therefore, more research is urged to investigate the potential distribution changes of wide‐ranged and narrow‐ranged species in response to climate change within a certain lineage.

The epiphytic orchid genus *Holcoglossum* Schltr. comprises 23 species with high conservation and ornamental values (POWO 2023), and distributes from northern Indochina Peninsula to Southern China (Xiang et al. 2012; Li et al. 2019). All *Holcoglossum* species are listed in the Convention on International Trade in Endangered Species of Wild Fauna and Flora (CITES) (http://cites.org/eng). All *Holcoglossum* species are epiphytic on tree trunks in broad‐leaved evergreen forests or rocks in forests, with the elevation above 700 m (Jin and Wood 2009). A range of vegetative and floral variation exist within *Holcoglossum*, such as short to long leaves (3–60 cm) and diverse flower colors (white, purple, pink, and yellow) (Chen et al. 2009). In addition, the pollination systems of *Holcoglossum* are also divergent, including autogamy, bee, ant, beetle, and bird (Xiang et al. 2012). The distributions of *Holcoglossum* species are distinctively different, such as *H. flavescens* with a wide range throughout southern China and *H. quasipinifolium* only in Taiwan (China). Fan et al. (2009) proposed that *Holcoglossum* could be divided into three clades: alpine clade (AC), tropical clade (TC), and the intermediate clade (HC), which was also supported in later research (Xiang et al. 2012; Zhao et al. 2020). Zhao et al. (2020) found that *Holcoglossum* originated in the Paleotropical region 8.53 million years ago (Ma), and diverged in situ since the late Miocene (6.33 Ma). Therefore, *Holcoglossum* was a key group to explore the potential distribution changes of wide‐ranged and narrow‐ranged species in response to the long‐term climate changes from the Pliocene to the future.

Currently, an increasing number of studies have used species distribution models (SDMs) to predict the potential distributions under climate changes of many plants (Gong et al. 2020; Fang et al. 2021), such as invasive Asteraceae plants (Yang et al. 2023) and orchid species *Prosthechea jauana* (Vieira et al. 2024), *Corybas taliensis* (Liu et al. 2024), *Habenaria* and *Calanthe* species (Qiu et al. 2023). Duan et al. (2014) indicated that compared with other models (such as Generalized Linear Model (GLM) Generalized Addition Model (GAM), and so on), the maximum entropy (MaxEnt) model had good prediction and stability. Wisz et al. (2008) also suggested that the MaxEnt model had better performance than other models for species with limited occurrence records. Therefore, we chose the MaxEnt model to predict the historical and future potential distributions of *Holcoglossum* species.

To explore the distribution changes of wide‐ranged and narrow‐ranged species in different climate scenarios, we used the MaxEnt model to predict the potential distributions of eight *Holcoglossum* species combined with the species' occurrence records, 19 climatic variables, and human footprint. The objects of this study include: (1) What are the key predictor variables in determining the potential distributions of wide‐ranged and narrow‐ranged *Holcoglossum* species? (2) How did the potential distribution changes of wide‐ranged and narrow‐ranged *Holcoglossum* species change from the Pliocene to the future? (3) What are the future migration directions of wide‐ranged and narrow‐ranged *Holcoglossum* species? This study aims to gain a better understanding of the future spatial distributions of *Holcoglossum* species, which is crucial for their future distributions and conservation under global warming.

## 2 Material and Methods

### 2.1 Species Distribution Data Assembly and Filtering

The occurrence records of *Holcoglossum* species were collected from the Global Biodiversity Information Facility (GBIF; http://www.gbif.org/), National Specimen Information Infrastructure (NSII; http://www.nsii.org.cn/), and Plant Photo Bank of China (PPBC; https://ppbc.iplant.cn/). After removing the occurrence records from misidentified specimens and those outside of their native ranges, we obtained a total of 183 occurrence records of 23 *Holcoglossum* species. Among them, eight *Holcoglossum* species with more than 10 occurrence records were selected for subsequent analyses. In order to exclude the influence of autocorrelation and uneven sampling intensity, we only reserved one occurrence record every 5 km in ArcGIS v10.8. Finally, 118 eligible records of eight *Holcoglossum* species were used for modeling (Table S1).

### 2.2 Predicted Variable Selection

Nineteen bioclimatic variables from the Last Glacial Maximum (LGM), the Last Interglacial (LIG), the present climate (representative of 1970–2000), and future climate data (for 2050, 2070, and 2090; with 2050 representing the average of 2041–2060, 2070 representing the average of 2061–2080, and 2090 representing the average of 2081–2100) were selected from the WorldClim v1.4 database (https://www.worldclim.org/) at 2.5‐arc‐min spatial resolution (Table S2). The future climate data (2050, 2070, and 2090) were derived from BBC‐CSM2‐MR and CMCC‐ESM2 models, respectively. The climate layers of 3.205 Ma (Pliocene) were derived from Brown et al. (2018). Under the BBC‐CSM2‐MR and CMCC‐ESM2 models, we selected three climate scenarios (the optimistic scenario SSP1‐2.6, the moderate scenario SSP2‐4.5, and the pessimistic scenario SSP5‐8.5), respectively. SSP1, SSP2, and SSP5 describe future pathways with low, medium, and high challenges for adaptation and mitigation, respectively (Popp et al. 2017). In addition, the 2022 Human Footprint data (HFP) was downloaded from Mu et al. (2022) and was used to predict the potential distributions of eight *Holcoglossum* species in the present and future. To avoid the distortion of MaxEnt model estimation caused by multicollinearity among the 19 bioclimatic variables, we excluded the bioclimatic variables with Pearson |*r*| > 0.8 (Wei et al. 2020; Gao et al. 2022) for each period using SPSS Statistics 26 (https://www.ibm.com/cn‐zh/spss/), respectively (Table S3).

### 2.3 Species Distribution Modeling

MaxEnt v3.4.3 (https://biodiversityinformatics.amnh.org/open_source/maxent/) was used to assess the relative importance of predictor variables and predict the potential distributions of eight *Holcoglossum* species in the Pliocene, LGM, LIG, present, and future (2050: SSP1‐2.6, SSP2‐4.5, and SSP5‐8.5; 2070: SSP1‐2.6, SSP2‐4.5, and SSP5‐8.5; 2090: SSP1‐2.6, SSP2‐4.5, and SSP5‐8.5). These processes were repeated 10 times using a cross‐validation procedure. The AUC values were divided as follows: 0.5–0.6 (poor), 0.6–0.7 (fair), 0.7–0.8 (good), 0.8–0.9 (very good), and 0.9–1.0 (excellent) (Swets 1988); the TSS values were divided as follows: 0–0.4 (poor), 0.4–0.5 (fair), 0.55–0.7 (good), 0.7–0.85 (very good), and 0.85–1.0 (excellent); the KAPPA values were divided as follows: 0–0.4 (poor), 0.4–0.5 (fair), 0.55–0.7 (well), 0.7–0.85 (very good), and 0.85–1.0 (excellent) (Lu et al. 2025). We classified the results of the MaxEnt model into four ranks (i.e., high suitable, medium suitable, low suitable, and unsuitable) using the “Reclassify tool” in ArcGIS v10.8 (https://www.esri.com/zh‐cn/arcgis/).

For eight *Holcoglossum* species, we calculated the potential distribution areas in the past and future using SDM toolbox v2.4 (Brown 2014), respectively. To further investigate the potential distribution changes of each species since the Pliocene to the future, we calculated the relative change ratio of potential distribution areas between two near periods. Finally, in order to predict the possible migration of eight *Holcoglossum* species from current to future climate scenarios, we used the “Centroid changes tool” in SDM toolbox v2.4 (Brown 2014) to analyze the migration of high suitable distribution centroids of each *Holcoglossum* species.

Many previous studies selected an appropriate threshold to make the division of wide‐ranged and narrow‐ranged species according to the current distribution area of each species (Geng et al. 2012; Yu et al. 2019). According to the current potential distribution areas based on the MaxEnt model, we divided the eight *Holcoglossum* species into wide‐ranged and narrow‐ranged with a threshold of 5000 km^2^ (Table S4). Specifically, *H. amesianum*, *H. flavescens* , *H. himalaicum*, and *H. subulifolium* were considered wide‐ranged species; *H. kimballianum*, *H. pumilum* , *H. quasipinifolium*, and *H*. *wangii* were narrow‐ranged species.

## 3 Results

### 3.1 Model Accuracy Evaluation

We compared the potential distribution areas of eight *Holcoglossum* species under the CMCC‐ESM2 and BBC‐CSM2‐MR models in 2090 (SSP1‐2.6, SSP2‐4.5, and SSP5‐8.5), and found that the results were very similar (Figure S1). Furthermore, Wu et al. (2019) considered that the BBC‐CSM2‐MR model represented higher accuracy in simulating global temperature, precipitation distribution, and atmospheric radiation. Therefore, we ultimately selected the BBC‐CSM2‐MR model for subsequent analyses.

Under the BCC‐CSM2‐MR model, we compared the potential distribution areas of the eight *Holcoglossum* species in three climatic scenarios (2090, 2070, and 2050), and found that the results were similar (Figure S2). Samset et al. (2020) pointed out that carbon dioxide can remain in the atmosphere for hundreds of years, and current emissions will continue to impact the climate for decades or longer. Therefore, we selected the 2090 (2081–2100) climate scenarios to better represent the potential distribution of *Holcoglossum* in the future.

We calculated the AUC, TSS, and KAPPA values of eight *Holcoglossum* species from the Pliocene to the future, and found that most values were above 0.85 (Tables S5–S7). These high AUC, TSS, and KAPPA values indicated high predictive performances (Landis and Koch 1977; Allouche et al. 2006; Phillips et al. 2006). Our results indicated the good performance and high accuracy of the model in this study.

### 3.2 Key Determining Variables of the Potential Distributions

**FIGURE 1:**
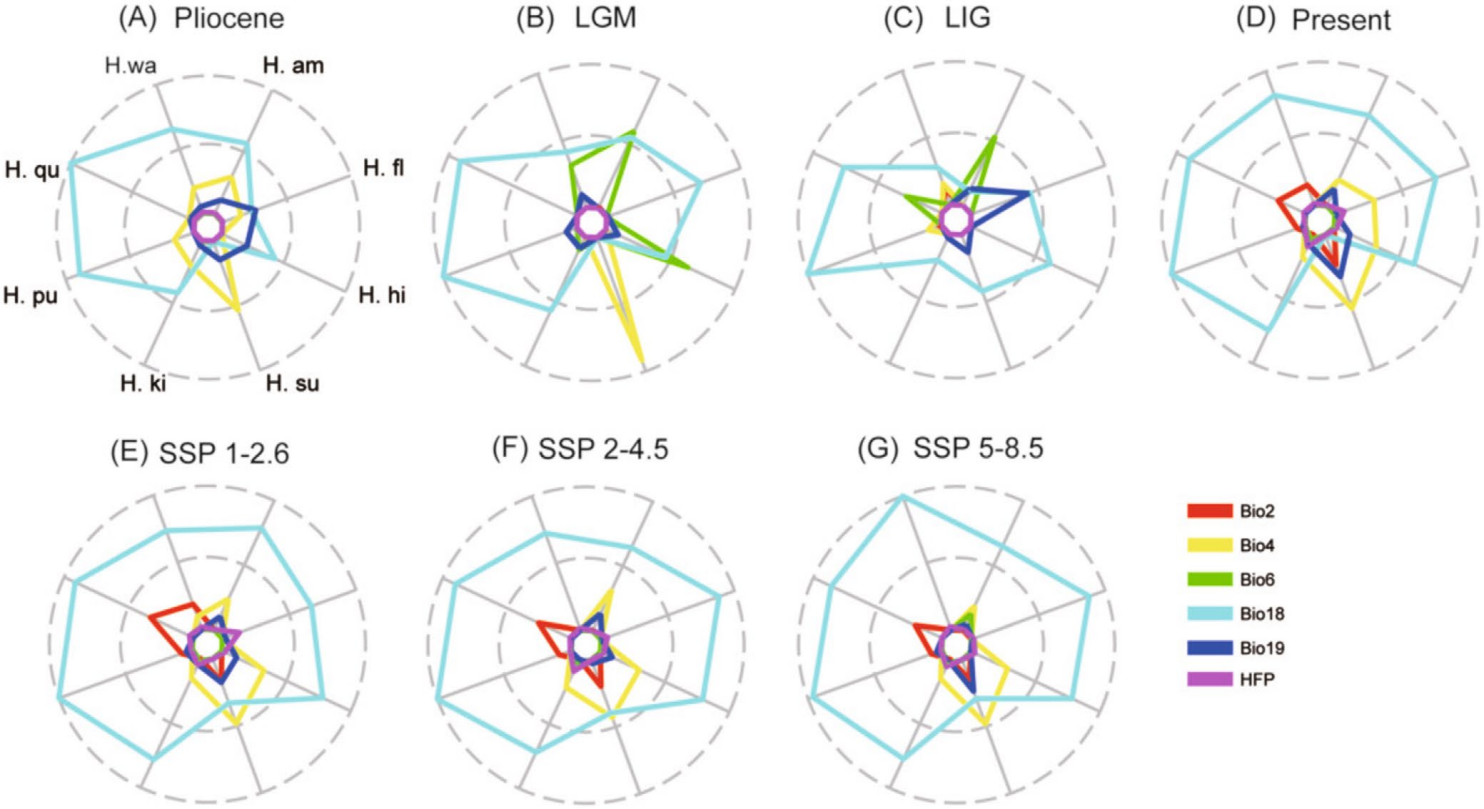
Contributions of bioclimatic variables for the historical and future potential distributions of eight wide‐ranged and narrow‐ranged *Holcoglossum* species. *H. am*, *H. amesianum*; *H. fl*, *H. flavescens* ; *H. hi*, *H. himalaicum*; *H. ki*, *H. kimballianum*; *H. su*, *H. subulifolium*; *H. pu*, *H. pumilum* ; *H. qu*, *H. quasipinfolium*; *H. wa*, *H. wangii*.

Our results showed that the distributions of eight *Holcoglossum* species were less relative to HFP in the future (Figure 1). For the wide‐ranged species, the precipitation of the warmest quarter (Bio18) was the main variable in determining the distributions of *H. amesianum* and *H. himalaicum*, whereas the precipitation of the coldest quarter (Bio19) and temperature seasonality (Bio4) were the main determining variables of *H. flavescens* and *H. subulifolium* in the Pliocene, respectively. As for the LGM, Bio18 and Bio4 were important in determining the potential distributions of *H. flavescens* and *H. subulifolium*, respectively, whereas the minimum temperature of the coldest month (Bio6) of *H. amesianum* and *H. himalaicum*. In the LIG, Bio18 was the main variable in determining the potential distribution of *H. flavescens* , *H. himalaicum*, and *H. subulifolium*, while Bio6 of *H. amesianum*. In addition, Bio18 was the main variable in determining the potential distributions of *H. amesianum*, *H. flavescens* , and *H. himalaicum*, whereas Bio4 of *H. subulifolium* in the present. For the future 2090 (SSP1‐2.6, SSP2‐4.5, and SSP5‐8.5), the main variable in determining the potential distributions of most wide‐ranged *Holcoglossum* species was Bio18, except for Bio4 of *H. subulifolium*.

For the narrow‐ranged species, Bio18 was always the main variable in determining the potential distributions from the Pliocene to the future (2090, SSP1‐2.6, SSP2‐4.5, and SSP5‐8.5).

### 3.3 Potential Distribution Under Different Climatic Scenarios

**FIGURE 2:**
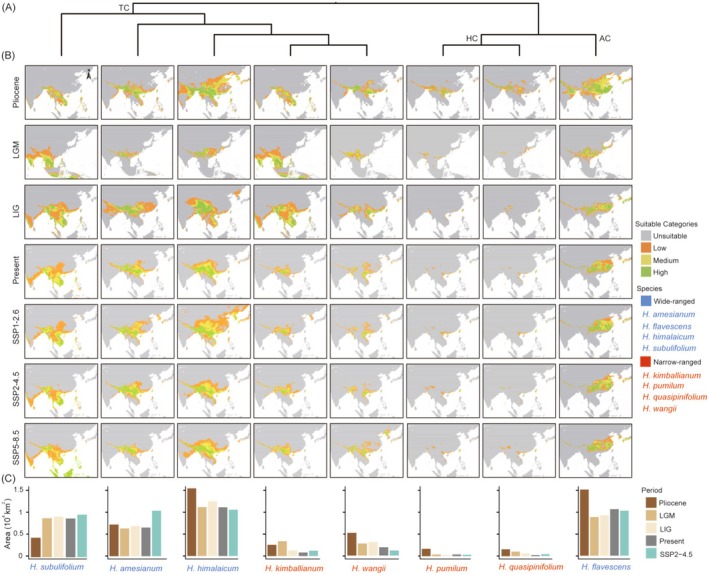
Potential distribution changes of eight wide‐ranged and narrow‐ranged *Holcoglossum* species under the BBC‐CSM2‐MR model. (A) Phylogeny tree of *Holcoglossum* modified from Zhao et al. (2020); (B) potential distribution changes from the Pliocene to the future 2090 (2081–2100) under three scenarios (SSP 1‐2.6, SSP 2‐4.5 and SSP 5‐8.5); (C) changes of potential distribution area of each species.

The potential distributions of the eight *Holcoglossum* species were shown in Figure 2. For the four wide‐ranged *Holcoglossum* species (except *H. subulifolium*), the potential distribution areas contracted from the Pliocene to the LGM. From the LGM to the LIG, the potential distribution areas of four wide‐ranged *Holcoglossum* species expanded. From the LIG to the present, the potential distribution areas of *H. amesianum*, *H. subulifolium*, and *H. himalaicum* contracted, whereas the other species expanded. From the present to 2090 (SSP2‐4.5), the potential distribution areas of *H. amesianum* and *H. subulifolium* expanded, whereas *H. flavescens* and *H. himalaicum* contracted.

For the four narrow‐ranged *Holcoglossum* species, the potential distributions of *H. pumilum* , *H. quasipinfolium*, and *H. wangii* contracted from the Pliocene to the LGM, whereas *H. kimballianum* expanded. From the LGM to the LIG, the potential distributions of the narrow‐ranged *Holcoglossum* species (except *H. wangii*) contracted. From the LIG to the present, the potential distributions of *H. kimballianum*, *H. quasipinfolium*, and *H. wangii* contracted, whereas *H. pumilum* expanded. From the present to the future (2090, SSP2‐4.5), *H. pumilum* and *H. wangii* contracted their potential distribution areas, whereas *H. quasipinfolium* and *H. kimballianum* expanded.

### 3.4 High Suitable Distribution Centroid Migration

**FIGURE 3:**
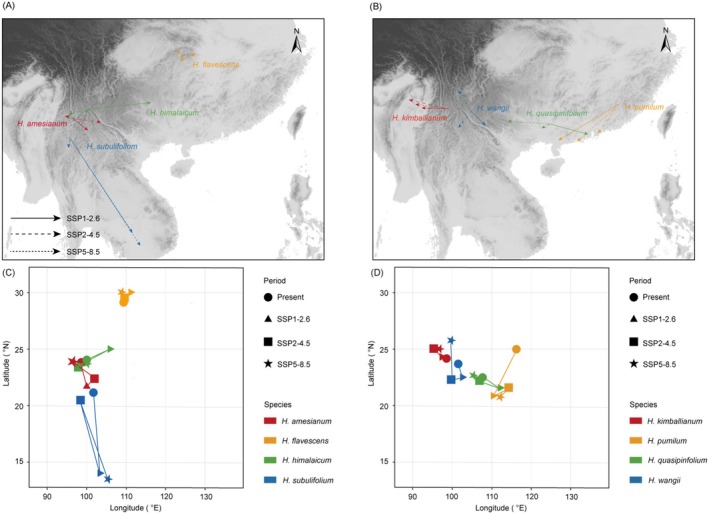
Centroid migration routes of eight wide‐ranged and narrow‐ranged *Holcoglossum* species in 2090 (SSP 1‐2.6, SSP 2‐4.5 and SSP 5‐8.5). (A, C) Wide‐ranged species; (B, D) narrow‐ranged species.

We further investigated the highly suitable distribution centroid migration of each *Holcoglossum* species from the present to the future (Figure 3; Table S8). For the four wide‐ranged *Holcoglossum* species, the centroids of *H. flavescens* and *H. himalaicum* had a tendency to shift eastwards in 2090 (SSP1‐2.6), while the other two had a tendency to shift southwards. In 2090 (SSP2‐4.5), the centroids of *H. amesianum*, *H. himalaicum*, and *H. subulifolium* would shift southwards, while *H. flavescens* would experience nearly no migration. In 2090 (SSP5‐8.5), the centroids of *H. amesianum* and *H. flavescens* had a tendency to shift westwards, whereas *H. subulifolium* and *H. himalaicum* had a tendency to shift southwards.

For the four narrow‐ranged species, centroids of *H. pumilum* , *H. quasipinfolium*, and *H. wangii* had a tendency to shift southwards in 2090 (SSP1‐2.6), while *H*. *kimballianum* shifted westwards. In 2090 (SSP2‐4.5), centroids of *H. quasipinfolium*, *H. pumilum* , and *H. wangii* shifted southwest, whereas *H. kimballianum* shifted westwards. In 2090 (SSP5‐8.5), the centroids of *H. kimballianum*, *H. quasipinfolium*, and *H. wangii* shifted westwards, and *H. pumilum* had a tendency to shift southwards.

## 4 Discussion

### 4.1 Potential Distribution Changes of Wide‐Ranged and Narrow‐Ranged Species

Precipitation of the warmest quarter was the key environmental factor in determining the potential distribution areas of seven *Holcoglossum* species (except *H. subulifolium*) in the future (Figure 1). Similarly, precipitation variables were the main factors of the potential distribution of other orchids, such as *Prosthechea jauana* (Vieira et al. 2024), *Dactylorhiza hatagirea* (Sharma et al. 2023), *Corybas himalaicus* (Tao et al. 2024), and *Prosthechea jauana* (Vieira et al. 2024). Additionally, *Holcoglossum* species are typically epiphytic (Chen et al. 2009), and precipitation was a key factor for the distribution of epiphytes (Zotz 2016; Taylor et al. 2022).

From the Pliocene to the present, the potential distribution areas of both wide‐ranged (except *H. subulifolium*) and narrow‐ranged species contracted in response to climate changes. From the present to the future, the potential distribution areas of two wide‐ranged and two narrow‐ranged species contracted, whereas the potential distribution areas of the others expanded (Figure 2; Table 1). Our results did not fully support the hypothesis that narrow‐ranged species were more vulnerable to climate change than wide‐ranged species. Indeed, other research also challenged the negative correlation between current distribution range and potential distribution change in response to the changing climate. For example, a recent study showed that 4 of 10 narrow‐ranged *Rhododendron* species would expand their potential distribution in the future, while the other six narrow‐ranged and 10 wide‐ranged *Rhododendron* species would contract (Yu et al. 2019). Similarly, Qiu et al. (2023) also indicated that species potential distribution changes in the future were not correlated with their current range sizes for two terrestrial orchid genera (*Habenaria* and *Calanthe*).

**TABLE 1:** Potential distribution area changes between different periods for eight wide‐ranged and narrow‐ranged *Holcoglossum* species.

Distribution range	Species	Pliocene‐LGM	LGM‐LIG	LIG‐Present	Present‐Future
Wide	*H. amesianum*	−5.14%	2.89%	−6.36%	65.58%
	*H. flavescens*	−41.62%	7.32%	10.29%	−1.89%
	*H. himalaicum*	−27.75%	11.77%	−2.85%	−12.85%
	*H. subulifolium*	103.49%	4.35%	−2.76%	7.48%
Narrow	*H. kimballianum*	43.49%	−43.06%	−24.36%	32.45%
	*H. pumilum*	−66.84%	−74.50%	13.10%	−7.80%
	*H. quasipinfolium*	−68.54%	−62.84%	−40.22%	16.51%
	*H. wangii*	−35.92%	10.45%	−36.54%	−27.62%

Our results also revealed the close relationships between climate factors and the potential distribution areas of *Holcoglossum* species. From the Pliocene to the present, 50% of species always were affected by Bio18. Seven species were affected by Bio18 except *H. subulifolium* from the present to the future (SSP1‐2.6, SSP2‐4.5, and SSP5‐8.5) (Figure 1). Djordjević et al. (2016) found that the distributions of 42 orchid taxa in western Serbia were related to climate factors. With climate change (whether it is warming or cooling), the potential distribution areas of both wide‐ranged and narrow‐ranged species would contract or expand simultaneously, regardless of their current distribution range. This might be due to the fact that the current distribution area only represents the adaptability under the current climate, while the adaptability to new environments varies specifically after climate changes. Therefore, we speculated that the vulnerability to climate change of species might be unlinked to the current distribution range.

### 4.2 The Relationship Between Potential Distribution Area Changes and Phylogeny

To investigate whether closely related species tend to present similar potential distribution changes in response to climate change, we combined the potential distribution changes of eight *Holcoglossum* species and the time‐calibrated phylogenetic tree from Zhao et al. (2020) and found that the potential distribution changes of the species in the same clades did not show the same trend in response to the long‐term climate change from the Pliocene to the future (Figure 2; Table 1). In the HC clade of *Holcoglossum*, the potential distribution area of *H. pumilum* contracted from the Pliocene to the future, whereas the potential distribution area of *H. quasipinifolium* contracted from the Pliocene to the present and expanded in the future (Figure 2, Table 1). Previous studies indicated that due to phylogenetic niche conservatism (Wiens and Graham 2005), closely related species tend to perform more similarly in niche requirements than less related species (Losos 2008; Holt 2009).

In the TC clade of *Holcoglossum*, Bio18 was the main variable in determining the distributions of species in the future (2090, SSP2‐4.5), while the other influencing environmental factors were different from each other. Bio4 was the second most important environmental factor for *H. amesianum*, *H. subulifolium*, and *H. kimballianum*, and the distributions of the three species tended to expand. However, Bio4 was also the second most important environmental factor for *H. himalaicum*, and this species tended to contract (Figures 1 and 2). Furthermore, Bio7 was the second most important environmental factor for *H. wangii*, and this species tended to contract. Lawlor et al. (2024) proposed that different climate factors might have an impact on potential distribution area changes. Similar results were obtained in the study of *Bergenia* (Qiu et al. 2024). The main environmental factor of *Bergenia ciliata* was the precipitation of the driest quarter (Bio17), which caused the potential distribution areas to expand; and the main environmental factor of *Bergenia purpurascens* was Bio18, which caused the potential distribution areas to contract (Qiu et al. 2024).

Additionally, the vegetative and floral characters of *Holcoglossum* species were diverse, especially the divergent pollination systems (Chen et al. 2009; Xiang et al. 2012). These characters may play roles in the distribution changes of *Holcoglossum* under climate change in the future. Thus, it is better to further explore the relationships between the biological characters and the potential distribution areas in the future.

### 4.3 Implications for *Holcoglossum* Conservation

Our results indicated that five of eight *Holcoglossum* species migrated to lower latitudes in the future (Figure 3). Although the vulnerability of species to future climate change was not directly linked to their current distribution range, we found that the potential distribution areas of narrow‐ranged species were relatively small and fragmented (Figure 2). With the drastic climate changes from the Pliocene to the future, the high suitable distributions for *Holcoglossum* species were always concentrated in the East Asian mainland (Figure 2). Furthermore, the potential distribution areas of four species contracted ( *H. flavescens* , *H. himalaicum*, *H. pumilum* , and *H*. *wangii*). Therefore, it is necessary to pay more attention to the protection of species whose potential distribution areas contract in the future. Our findings are of great significance for the conservation of epiphytic orchids.

## 5 Conclusions

In this study, we found the key environmental factor in determining the potential distributions of seven *Holcoglossum* species was the precipitation of the warmest quarter (expect *H. subulifolium*) in the future. We speculated that the vulnerability to climate change of species might be unlinked to the current distribution range and phylogeny. The centroids of three wide‐ranged species would migrate southwards (*H. amesianum*, *H. himalaicum*, and *H. subulifolium*), whereas *H. flavescens* had nearly no migration; the centroids of three narrow‐ranged species would migrate southwards ( *H. pumilum* , *H. quasipinifolium*, and *H. wangii*), while *H. kimballianum* was predicted to migrate westwards. This research provided insights to understanding the potential distribution changes of epiphytic orchids and the conservation of wide‐ranged and narrow‐ranged orchid species in response to climate change.

## Author Contributions

**Pei‐Yang Zhang:** data curation (equal), formal analysis (equal), visualization (equal), writing – original draft (equal). **Peng Zhou:** data curation (equal), investigation (equal), writing – review and editing (equal). **Yi‐Zhen Liu:** resources (equal), writing – review and editing (equal). **Zhi‐Wen Xu:** resources (equal), writing – review and editing (equal). **Lei‐Qiang Gong:** resources (equal), writing – review and editing (equal). **Xiao‐Guo Xiang:** conceptualization (equal), funding acquisition (equal), project administration (equal), resources (equal), supervision (equal), writing – review and editing (equal).

## Conflicts of Interest

The authors declare no conflicts of interest.

## Supporting information


Data S1

Figure S1

Figure S2

Table S1

## Data Availability

All data used in the study are included in this paper and Supporting Information.
