# Supplementary material for: Distribution Dynamics of Wide‐Ranged and Narrow‐Ranged Species From the Pliocene to the Future: Insights From Asian Endemic Holcoglossum (Orchidaceae)
Source: Ecol Evol. 2025 Apr 14;15(4):e71301. doi: 10.1002/ece3.71301 (PMC11994890; doi:10.1002/ece3.71301)
Supplement: Supplementary file 1 — Data S1 [file ECE3-15-e71301-s002.docx]

**Supplementary Information**

**Table S1. Occurrence records of eight wide-ranged and narrow-ranged *Holcoglossum* species used in species distribution modeling.**

**Table S2. Environmental and Human Footprint variables used in the species distribution modeling of eight wide-ranged and narrow-ranged *Holcoglossum* species.**

**Table S3. Pearson |r| values of eight wide-ranged and narrow-ranged *Holcoglossum* species from Pliocene to future climate scenarios (2090: 2081–2100).**

**Table S4. High suitable distribution areas of eight wide-ranged and narrow-ranged *Holcoglossum* species under current climate condition.**

**Table S5. List of AUC values in MaxEnt model for eight wide-ranged and narrow-ranged *Holcoglossum* species.**

**Table S6. List of TSS values in MaxEnt model for eight wide-ranged and narrow-ranged *Holcoglossum* species.**

**Table S7. List of KAPPA values in MaxEnt model for eight wide-ranged and narrow-ranged *Holcoglossum* species.**

**Table S8. The coordinates of the highly suitable distribution centroids of eight wide-ranged and narrow-ranged *Holcoglossum* species from present to future climate scenarios.**

**Figure S1. Potential distribution changes of eight wide-ranged and narrow-ranged *Holcoglossum* species under the CMCC-ESM2 model**. (A) Phylogeny tree of *Holcoglossum* was modified from Zhao et al. (2020); (B) Potential distribution changes in the future 2070 (2061–2080) under three scenarios (SSP 1-2.6, SSP 2-4.5 and SSP 5-8.5); (C) Potential distribution changes in the future 2090 (2081–2100) under three scenarios (SSP 1-2.6, SSP 2-4.5 and SSP 5-8.5).

**Figure S2. Potential distribution changes of eight wide-ranged and narrow-ranged *Holcoglossum* species under the BBC-CSM2-MR model.** (A) Potential distribution changes in the future 2050 (2041–2060) under three scenarios (SSP 1-2.6, SSP 2-4.5 and SSP 5-8.5); (B) Potential distribution changes in the future 2070 (2061–2080) under three scenarios (SSP 1-2.6, SSP 2-4.5 and SSP 5-8.5). The phylogeny tree and taxon orders were consistent with Figures 2 and S1.
