# Supplementary figures and images for: Distribution Dynamics of Wide‐Ranged and Narrow‐Ranged Species From the Pliocene to the Future: Insights From Asian Endemic Holcoglossum (Orchidaceae)
Source: Ecol Evol. 2025 Apr 14;15(4):e71301. doi: 10.1002/ece3.71301 (PMC11994890; doi:10.1002/ece3.71301)

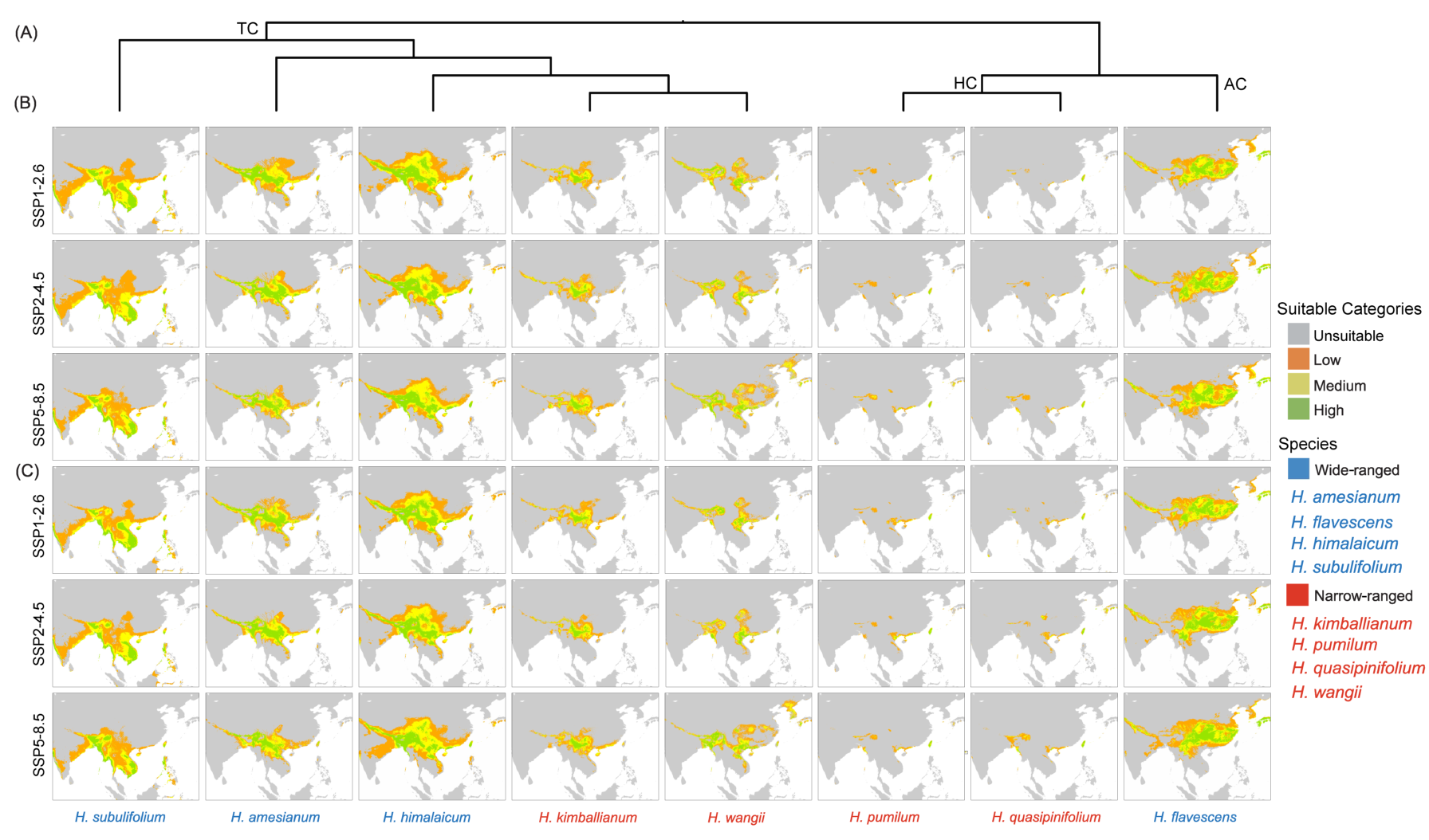

Supplement: Supplementary file 2 — Figure S1 [file ECE3-15-e71301-s001.tif]

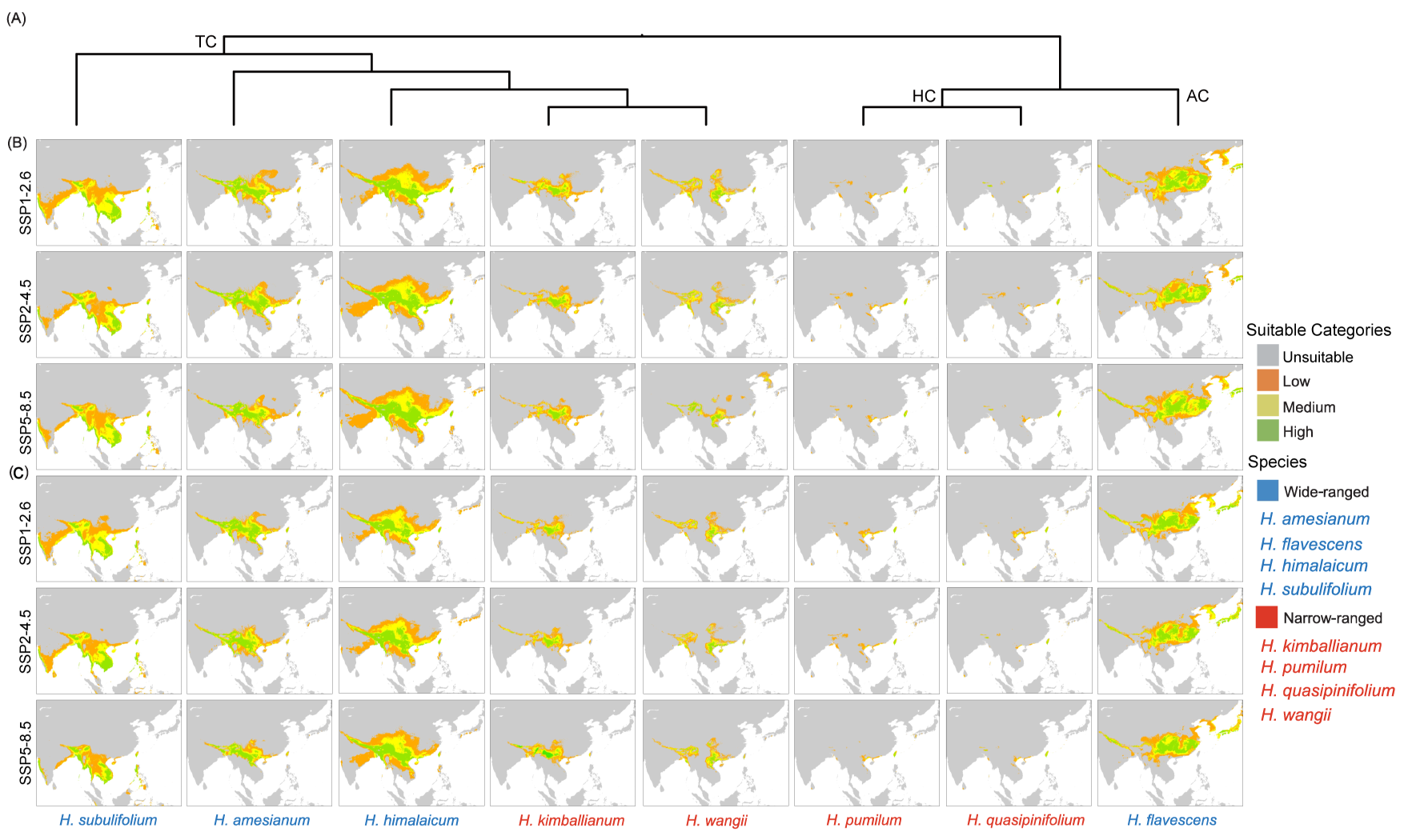

Supplement: Supplementary file 3 — Figure S2 [file ECE3-15-e71301-s003.tif]
